# Efficacy and Safety of Thoracic Epidural Analgesia in Patients With Acute Pancreatitis: A Narrative Review

**DOI:** 10.7759/cureus.23234

**Published:** 2022-03-16

**Authors:** Abhijit Nair, Manish Kumar Tiwary, Suresh Seelam, Krishna Kishore Kotthapalli, Kaushik Pulipaka

**Affiliations:** 1 Anesthesiology, Ibra Hospital, Ibra, OMN; 2 Anesthesiology, Royal Hospital, Muscat, OMN; 3 Anesthesiology, Ibri Hospital, Ibra, OMN; 4 Anesthesia and Critical Care, Sultan Qaboos University Hospital, Muscat, OMN

**Keywords:** neuraxial analgesia, severe acute pancreatitis, intensive care unit, thoracic epidural analgesia, acute pain management

## Abstract

Patients admitted to the intensive care unit with moderate to severe acute pancreatitis carry significant morbidity and mortality. A few unfortunate patients in whom the initial line of treatment fails to show clinical improvement develop multiorgan dysfunction involving lungs (adult respiratory distress syndrome), renal failure, intra-abdominal infections, sepsis, and septic shock, which ultimately leads to prolonged hospitalization and a substantial cost of treatment. The acute abdominal pain experienced by these patients is excruciating and requires multimodal analgesia. Continuous epidural analgesia has been found to provide good quality, opioid-sparing analgesia in these patients. A few studies have also demonstrated that segmental sympathectomy resulting from epidural blockade could lead to lowering of serum amylase and lipase levels improve paralytic ileus, and thus hastens the process of recovery. The present paper aims to discuss the advantages of continuous epidural analgesia in patients with acute pancreatitis of varying severity and to review the existing literature using specific keywords.

## Introduction and background

Acute pancreatitis is a medical condition that has high morbidity and mortality in moderate to severe situations. The present global incidence is in the range of 5-30 per 100,000 population per year [1]. Based on the Revised Atlanta classification, acute pancreatitis is classified as mild, moderately severe, or severe acute pancreatitis [2]. The moderately severe and severe acute pancreatitis patients carry significant morbidity and mortality as it is associated with multi-organ dysfunction like acute respiratory distress syndrome (ARDS), renal failure, prolonged intensive care unit (ICU) stay, and leads to a substantial cost in treatment [3]. The management includes goal-directed fluid resuscitation, enteral feeding, analgesia, and using appropriate scoring systems for prognostication [4,5]. The commonest causes of acute pancreatitis are gall stones, alcoholism, hypertriglyceridemia, and drug-induced (estrogens, sulfonamides, tetracycline, azathioprine, valproate, methyldopa, corticosteroids, frusemide, etc.). The other causes are autoimmune diseases, secondary to infections (viral, bacterial, parasitic), secondary to interventional procedures (endoscopic retrograde cholangiopancreatography), postoperative, hypercalcemia, toxins, trauma, smoking, congenital causes like the annular pancreas, genetic disorders, vasculitis, and otherwise idiopathic [6].

Patients with acute pancreatitis present with epigastric pain of variable intensity depending on the severity, and are usually associated with nausea and vomiting. The etiology of pancreatitis also decides the type of pain. In biliary diseases, the patient describes a sharp pain radiating through to the back with more of acute onset. In alcoholics, the pain is often described as dull and generalized [7,8].

Pathophysiology

In acute pancreatitis, there is dysfunction of the pancreatic acinar cells, resulting from inappropriate activation of trypsinogen which is the proenzyme of trypsin, in its active form trypsin. This active trypsin activates trypsinogen in the pancreas, leading to an accumulation of trypsin in the acinar cells followed by an inflammatory cascade which leads to the damage of the pancreas. This ongoing inflammation releases pro-inflammatory cytokines interleukin (IL)-1, IL-6, and IL-8 and systemic mediators such as tumor necrosis factor-alpha (TNF-α) leading to systemic inflammatory response syndrome (SIRS) [9].

The activated sympathetic nervous system releases excessive catecholamines in the blood circulation which reduces the blood flow to the visceral organs and also interferes with pancreatic perfusion per se. Severe acute pancreatitis manifests in two phases, namely early and late phases. In the initial 10 days of the early phase, there is hypovolemia, SIRS, leaky capillaries, ascites, paralytic ileus, multi-organ involvement especially renal dysfunction. The severity of abdominal pain in the early phase is more and needs to be managed using multi-modal analgesia. The drugs used are opioids, non-steroidal anti-inflammatory drugs (NSAIDs), and thoracic epidural analgesia (TEA) in a few studies [10]. In the late phase, the problems encountered are pneumonia, bacteremia, intra-abdominal infections due to pancreatic necrosis, intra-abdominal collections, pseudocyst formation which increases the morbidity and mortality significantly [11]. The only definitive treatment in acute pancreatitis is when the etiology is biliary stones which are managed surgically or with endoscopic procedures. In other situations, the management is supportive with intravenous (IV) fluids, correction of electrolytes, enteral feeding, analgesia, and managing organ dysfunction due to SIRS [12].

There are several modalities available now to offer pain relief after major abdominal surgeries like transversus abdominis plane block, rectus sheath block, quadratus lumborum block, erector spinae plane block, continuous wound infiltration with local anesthetics (LA), and opioid-sparing multimodal analgesia, which either alone or in combination have been effective with variable efficacy. However, thoracic epidural analgesia remains the gold standard for providing good quality postoperative analgesia after major abdominal surgeries by facilitating early bowel activity, lesser incidence of postoperative pneumonia, lesser postoperative nausea/vomiting (PONV), and opioid use [13,14]. Although the Enhanced Recovery After Surgery (ERAS) society recommendations suggest continuous wound infiltration through a pre-peritoneal catheter as a viable alternative to TEA, the advantages of TEA cannot be denied or ignored [15,16].

## Review

There are different types of review articles including guidelines from various societies which have published evidence-based management of acute pancreatitis. This includes imaging modalities, intravenous hydration, enteral nutrition, surgical intervention, prevention of multi-organ dysfunction, and use of antibiotics in such situations. However, to date, the literature lacks guidelines or recommendations for providing good-quality, opioid-sparing analgesia in patients admitted with acute pancreatitis to the ICU. TEA has been hypothesized as an attractive, opioid-sparing analgesic modality in such situations by many researchers [17,18].

Effect of TEA on regional circulation in sepsis

It is well known that in sepsis, there is splanchnic hypoperfusion leaving the gut with lesser blood flow. This jeopardizes the integrity of gut mucosa which leads to bacterial translocation that has deleterious consequences often deciding the outcome of a critically ill patient admitted with sepsis [19]. TEA leads to a segmental sympathetic block which is targeted in such a way that it extends to the neural supply of splanchnic circulation, the compromised blood flow will be restored and will thus avoid serious events. There is a serious dearth of human data to explore this hypothesis [20]. However, Daudel et al. investigated the efficacy of TEA in rats with sepsis to improve gut mucosal microcirculation. The findings were encouraging but this did not translate into well-designed human research because of adverse hemodynamic and infective consequences of initiating TEA in patients with established sepsis [21].

Studies involving human participants

Medline, PubMed, Web of Science, Scopus, and Cochrane databases were searched using keywords: Acute Pain, Analgesia, Epidural, Pain management, Pancreatitis, to identify articles that described the use of epidural analgesia in patients with acute pancreatitis of various severity. To date, there is a dearth of a well-designed, adequately powered, randomized-controlled trial that investigated the efficacy of TEA as an analgesic modality for patients with acute pancreatitis. Table 1 summarises the studies published to date, including a study protocol whose results are eagerly awaited.

**Table 1:** Summary of all human studies which investigated the efficacy of epidural analgesia in acute pancreatitis

Authors/year	Number of patients	Type of study	Primary outcome	Conclusions
Niesel et al (1991) [22]	26	Prospective	The effect of a fractional epidural blockade on acute pancreatitis	With epidural analgesia, amylase and lipase levels dropped significantly in 4 days of initiation. There were no infective or neurological complications. All patients were eventually discharged from the hospital.
Bernhardt et al (2002) [23]	121	Prospective	To demonstrate the effectiveness and safety of epidural anaesthesia in patients with severe acute pancreatitis	Epidural analgesia was tolerated well with acceptable hemodynamic fluctuations
Sadowski et al (2015) [24]	35	Randomized trial	To investigate the safety of epidural anesthesia, its effect on pancreatic perfusion (using computed tomography) and the outcome of patients with acute pancreatitis	Epidural analgesia increased arterial perfusion of the pancreas and improved the clinical outcome of patients with acute pancreatitis
Sasabuchi et al (2017) [25]	307	Retrospective cohort study	To describe the characteristics, morbidity and mortality of patients with acute pancreatitis treated with epidural analgesia	Although the efficacy of epidural analgesia in the management of pain in patients with acute pancreatitis appears effective, epidural analgesia is not widely used for analgesia.
Bulyez et al (2017) [28]	148	Study protocol	To investigate whether epidural analgesia reduced acute pancreatitis-associated respiratory failure and other major clinical outcomes	Results have not been published yet
Jaboudon et al (2018) [26]	1003	Multicentre propensity analysis	To assess the impact of epidural analgesia on mortality in ICU patients with acute pancreatitis	Mortality at 30 days was lower in patients who received epidural analgesia than in patients who did not.
Tyagi et al (2019) [27]	32	Randomized trial	To evaluate the effect of thoracic epidural block on progression of acute pancreatitis induced organ dysfunction/failure	Thoracic epidural was associated with insignificant clinical trend towards better organ functions and lesser mortality with a significant greater lowering of serum procalcitonin.

In 1991, Niesel et al. published a study involving 26 patients to investigate the effect of fractional epidural in patients with acute pancreatitis [22]. Twenty patients received thoracic epidural and six patients were managed with lumbar epidural analgesia. The duration of epidural analgesia varied from one to 15 days. The authors noticed that serum lipase levels reduced from 8120 to 427 IU, and alpha-amylase levels reduced from 1401 to 143 IU. In this series, all patients had a successful outcome and were discharged from the hospital eventually.

In 2002, another study by Bernhardt et al. described the successful use of epidural analgesia in 121 patients with severe acute pancreatitis. Out of 1496 observation days, excellent analgesia was documented in 1083 days (72%) without the use of any other systemic analgesia [23]. The average duration required for the normalization of serum amylase and lipase was 17.4 days. Three patients (2.5%) died who were having grade III acute pancreatitis. Overall, epidural analgesia was tolerated well with acceptable hemodynamic fluctuations.

Sadowski et al. randomized 35 patients predicted to have acute pancreatitis to receive TEA versus patient-controlled analgesia (PCA). Thirteen patients received TEA and 22 received PCA [24]. The median duration of EA was 5.7 days with no infective or neurological complications. On analysis, the authors noticed more improvement in pancreatic perfusion in the TEA group (43%) than in the control group (7%). Pain scores were significantly better in the EA group than the control group (p=0.034 at 10 days). However, demography, length of hospital stays, and mortality were comparable in both groups.

Sasabuchi et al. conducted a retrospective cohort study that comprised 307 (0.7%) patients out of 44,146 patients discharged with acute pancreatitis who received TEA as an analgesic modality [25]. The median duration of TEA was four days (IQR, 3-5) with no complications like an abscess or hematoma. Six patients died during the hospital stay. The authors made an important point with this study that TEA is not commonly utilized as an analgesic modality in acute pancreatitis.

In a multicenter, propensity analysis by Jaboudon et al., 1003 patients with acute pancreatitis who received TEA as an intervention for analgesia were compared with patients who did not receive TEA [26]. A total of 46 patients received TEA and 212 patients died in 30 days. The authors used propensity score analysis to evaluate the risk of all-cause 30-day mortality in patients with acute pancreatitis receiving TEA (the years 2009-2014). They concluded that the all-cause 30-day mortality was significantly lower than that in matched patients who did not receive epidural analgesia (2% vs. 17%; p = 0.01).

Tyagi et al. randomized 32 patients in which they predicted to have acute pancreatitis to receive segmental TEA (t8-9, T9-10 level) versus no TEA which received IV morphine targeted for pain score less than 4 [27]. On analysis, the authors found that although the aggregate Sequential Organ Failure Assessment (SOFA) score during the stay in the ICU was similar in both the groups, there was a trend of improvement in the epidural group versus the other. Duration of hospital stay, patients requiring mechanical ventilation, and mortality were comparable but fall in serum procalcitonin was significantly greater in the epidural group. Patients in the epidural group did not present with significant hemodynamic changes or catheter-related infections. The limitation was a small sample size considering that it was a preliminary study.

Bulyez et al. designed an investigator-initiated, prospective multicentric, randomized controlled two-arm trial with assessor-blinded outcome assessment in 11 intensive care units across France, Belgium, and Switzerland to investigate the effects of epidural analgesia (EA) on organ failure, mortality, and clinical outcomes in critically ill patients with acute pancreatitis [28]. The authors called it The Epidural Analgesia for Pancreatitis (EPIPAN) trial. According to the authors, as of 2017, this was the first clinical trial to investigate the hypothesis of benefits of TEA in acute pancreatitis. The EPIPAN study planned to recruit 148 patients into two groups: the TEA group which will receive patient-controlled EA (ropivacaine and sufentanil) and a control group. All patients in either group will receive supportive care as per existing protocols with the exception being the analgesic modality (as per randomization). The primary outcome mentioned is the number of ventilator-free days at day 30 and the secondary outcomes are organ failure and mortality, levels of markers of systemic inflammation, epithelial lung injury, and healthcare-associated costs. As per the clinicaltrials.gov database, the EPIPAN study (NCT02126332) has completed the recruitment and the actual study completion date has been mentioned as February 26, 2019 [29]. At the time of drafting this review, there was no article published mentioning EPIPAN study except the study protocol which has been described earlier. We need to wait to see the results of EPIPAN study which could further enlighten the clinicians regularly involved in managing patients with acute pancreatitis.

Animal studies

Although adequately powered, well-designed studies investigating the role of TEA in acute pancreatitis involving human patients are less, there are several in-vivo and in-vitro animal studies mainly pigs and rats to investigate the pros and cons. Freise et al. randomized 28 rats into four groups: sham group with no pancreatitis, pancreatitis with NaCl, pancreatitis with EA, pancreatitis with EA after seven hours [30]. The authors monitored villus blood flow, serum amylase, lactate, and interleukin-6, and pancreatic injury was scored histologically. On analysis, the authors concluded that the use of TEA reduced systemic inflammation and also improved survival. Although microcirculation was restored, the inflammatory markers were not affected with TEA. The study by Demirag et al. involved 19 rats which were divided into three groups: acute pancreatitis with no intervention, rats who received TEA (T7-9) in absence of pancreatitis, and rats with acute pancreatitis who received TEA [31]. The authors found that TEA led to partial restoration of microcirculatory flow and prevented the development of tissue necrosis with reduced systemic complications.

Lauer et al. randomized 21 rats into three groups: acute pancreatitis with no intervention, acute pancreatitis with TEA, and TEA without pancreatitis [32]. Along with arterial blood gas, hemodynamics, and exhaled nitric oxide, they also performed in vitro studies of receptor-dependent and receptor-independent pulmonary vasoconstriction using an isolated perfused lung model. There is a school of thought that acute pancreatitis impairs hypoxic pulmonary vasoconstriction (HPV) due to increased production of nitric oxide. On analysis they found that the use of TEA in rats with acute pancreatitis led to better oxygenation, better lactate levels, lesser expired nitric oxide thus preserving HPV which was comparable to rats who received TEA in absence of pancreatitis. Later, Freise et al. investigated the effects of TEA (L3-4) in rats on hepatic function in four groups: group 1- normal rats, group 2- rats with TEA without necrotizing pancreatitis, group 3- rats with pancreatitis treated with saline, group 4- rats with pancreatitis and received TEA [33]. On analysis, they found that the use of TEA reduced liver injury in rats with necrotizing pancreatitis when compared to placebo.

Bachmann et al. randomized 34 pigs with induced severe acute pancreatitis to receive TEA in group one and no intervention in the other group [34]. After six hours, the authors measured and compared tissue oxygen tension (tpO2) in the pancreas and pancreatic microcirculation in pigs of both groups. On analysis, they concluded that TEA led to improved survival, enhanced microcirculatory perfusion, and improved tissue oxygenation with less histopathologic tissue damage in the TEA group. Winsö et al. randomized 16 pigs with induced acute pancreatitis into two groups: group 1- no intervention, group 2- TEA to investigate the efficacy of TEA to reduce insulin resistance and inflammatory response in presence of acute pancreatitis [35]. Blood sugar levels, serum insulin concentration, serum lipase, and relative tissue oxygen tension (PtiO2) levels were compared in both groups. On analysis, the authors concluded that TEA attenuated the progression of acute pancreatitis with acceptable glycemic levels and hemodynamics. The pigs in the TEA group had attenuated insulin resistance and fewer local pathophysiological events.

Discussion

TEA provides a targeted and segmental sympathectomy in a particular area, which leads to splanchnic vasodilatation and an improvement in local microcirculation (improved splanchnic and pancreatic perfusion, improved pancreatic microcirculation, reduced liver damage, and significantly reduced mortality). Segmental sympathetic blockade due to TEA is dose-dependent, site-dependent, and also dependent on the extent of the block [36]. Hypotension resulting from initiating TEA is a major hurdle in these patients as they already have SIRS which in many cases already leads to borderline systemic pressures [37]. However, this can be managed by goal-directed fluid therapy and at times by using vasopressors (norepinephrine) if necessary [38].

As concluded from animal studies, TEA confers protection by not only increasing pancreatic perfusion, it also improved the functioning of the liver, lungs, and intestines. Although the analgesic efficacy of TEA in acute pancreatitis is acceptable to many clinicians, the beneficial systemic effects conferred due to reduction of inflammatory markers, preservation of gut function is still anecdotal. The role of microcirculation in preventing pancreatic necrosis has been emphasized. Impaired microcirculation reduces the blood flow to the pancreas, interferes with healing, leads to increased vascular permeability, and causes intravascular thrombus formation. Pancreatic cells are very sensitive to hypoxemia and ischemia which if not addressed promptly can progress to severe pancreatitis which has detrimental outcomes. Impaired microcirculation due to ongoing inflammation progresses to necrotizing pancreatitis which has grave outcomes [39-41].

There are several limitations in this review. This is a narrative review and not a systematic review. We have discussed all published articles (human and animal studies) and presented the salient features in a tabular format. At this moment there is a dearth of prospective, randomized-controlled trials which explored analgesia efficacy and other pertinent advantages of TEA with the standard of care. Therefore, a statistical analysis was not done. Well-designed, multicentric studies with meticulous follow-up could answer this research question in the future. Moreover, the results of EPIPAN study are very much awaited.

## Conclusions

In patients with acute pancreatitis, the use of TEA appears to be a feasible option provided patient selection is appropriate and adequate hemodynamic monitoring is initiated to avoid unwanted adverse events like hypotension and infection. Many unanswered questions warrant research in the future. The information currently lacking is the timing for initiating TEA, level of TEA catheter placement, the optimal concentration of LA, the ideal LA for TEA, use of adjuvant in LA, the duration of TEA, continuous versus bolus analgesia versus patient-controlled analgesia (PCA). The hypothesis that the use of TEA facilitates recovery from the acute insult along with analgesia also needs to be explored with well-designed studies.
